# Age-severity matched cytokine profiling reveals specific signatures in Covid-19 patients

**DOI:** 10.1038/s41419-020-03151-z

**Published:** 2020-11-06

**Authors:** Roberta Angioni, Ricardo Sánchez-Rodríguez, Fabio Munari, Nicole Bertoldi, Diletta Arcidiacono, Silvia Cavinato, Davide Marturano, Alice Zaramella, Stefano Realdon, Annamaria Cattelan, Antonella Viola, Barbara Molon

**Affiliations:** 1 Fondazione Istituto di Ricerca Pediatrica - Città della Speranza, Padova, Italy; 2 Department of Biomedical Sciences, University of Padova, Padova, Italy; 3 Istituto Oncologico Veneto- IOV-IRCCS, Padova, Italy; 4 Infectious Disease Unit, Padova University Hospital, Padova, Italy; 5 Department of Medicine, Nephrology, Dialysis and Transplantation Unit, University of Padova, Padova, Italy; 6 Department of Surgery, Oncology and Gastroenterology, University of Padova, Padova, Italy

**Keywords:** Predictive markers, Translational research

## Abstract

A global effort is currently undertaken to restrain the COVID-19 pandemic. Host immunity has come out as a determinant for COVID-19 clinical outcomes, and several studies investigated the immune profiling of SARS-CoV-2 infected people to properly direct the clinical management of the disease. Thus, lymphopenia, T-cell exhaustion, and the increased levels of inflammatory mediators have been described in COVID-19 patients, in particular in severe cases^1^. Age represents a key factor in COVID-19 morbidity and mortality^2^. Understanding age-associated immune signatures of patients are therefore important to identify preventive and therapeutic strategies. In this study, we investigated the immune profile of COVID-19 hospitalized patients identifying a distinctive age-dependent immune signature associated with disease severity. Indeed, defined circulating factors - CXCL8, IL-10, IL-15, IL-27, and TNF-α - positively correlate with older age, longer hospitalization, and a more severe form of the disease and may thus represent the leading signature in critical COVID-19 patients.

## Introduction

The pathophysiology of COVID-19 is still a matter of scientific investigation. The disease seems to manifest itself in more or less severe forms depending on the age of the patients, with older people being at higher risk of developing serious complications^3^.

A timely coordinated host immune response represents the leading driver for restraining SARS-CoV-2 infection, having a remarkable impact on patient clinical outcomes. Indeed, several studies have appointed a dysregulated immunity as the crucial determinant for the failure of viral control.

An increased level of pro-inflammatory cytokines has been detected in patients with pulmonary inflammation and extensive lung damage^4^. In this regard, markedly high levels of interleukin IL-2R, IL-6, IL-10, and TNF-α have been reported in patients with severe illness^5^, although other reports suggest that more cytokines are involved in the COVID-19 pathogenesis^6,7^.

In addition to the deregulated cytokine response, COVID-19 patients show immunological alterations of the cellular compartment. Decreased total lymphocyte counts, T cell exhaustion, and defective lymphocyte responses were reported in patients^8–10^, suggesting that maladaptive immunity tips the balance between effective immune responses and unsolved inflammation in COVID-19 patients. In this scenario, the definition of novel clinical approaches aimed at re-direct and boost a proper, protective immune response should be considered.

The identification of early indicators of disease severity might improve the clinical management of COVID-19 patients having a great impact on the diagnostic and therapeutic decision making. Moreover, a more detailed picture of the immunological alterations characterizing different patients’ groups might offer novel insights for understanding COVID-19 pathogenesis.

To date, it is unclear whether specific immune signatures are associated with the severity of the disease at different patients’ ages.

In this study, we analyzed the cytokine and leukocyte profile of COVID-19 patients at hospital admission and identified distinctive immunological signatures that characterize younger or older severe patients. We found that severe patients under the age of 60 do not show major leukocyte alterations and express high levels of IL-1RA, IL-6, CCL2, CXCL1, CXCL9, CXCL10, and EGF. In contrast, older patients express high levels of CXCL8, IL-10, IL-15, IL-27, and TNF-α, present a significant reduction in the total T lymphocyte number and an increased expression of T cell exhaustion markers as compared to the younger.

These results provide novel manageable criteria to improve patient stratification at hospital entry and unveil novel age-dependent immune features of COVID-19-associated morbidity.

## Results and discussion

We evaluated the hospitalization time (HT), immune, and clinical features in a cohort of 44 *SARS*-*CoV*-*2*- positive symptomatic patients who have been admitted at the University Hospital of Padova from 9.04.2020 to 5.05.2020. The demographic and clinical data of patients are reported in Table 1. We selected three parameters that may be relevant to stratify COVID-19 patients, such as patients’ age, HT, and disease severity (DS) as defined by the WHO guidelines^11^.

**Table 1:** Demographic and Clinical data of COVID-19 patients at the hospitalization admission.

	All patients	Group 1	Group 2	*P*-value
	*N* = 44	(age < 60)	(age > 60)	
		%	%	
Gender, M/F	22/22	11/13	11/9	0.544827
Median Age, years (IQR)	57.5 (45–75)	48 (34–55)	76 (67–85)	***<0.001***
Diseases classification:				
Mild	38.65	54.1	20	***0.0304***
Moderate	20.45	16.66	25	0.7095
Severe	22.72	16.66	30	0.4716
Critical	18.18	12.50	25	0.4361
Symptoms at admission:				
Fever	84.09	87.5	80.9	0.6839
Cough	65.9	62.5	70	0.752
Dyspnoea	20.45	8.3	35	0.0573
Oxygen therapy	56.81	37.5	80	***0.0064***
Comorbidities ≥ 1(%)	79.5	62.5	100	***0.0021***
Smoking	20.45	33.33	5	***0.0271***
Alcohol consumption	2.27	4.1	0	1
HT, days Median (IQR)	12 (5–17.5)	6.5 (4.2–12)	15 (12–24.5)	***0.0082***

*P* values (two-sided) were computed using Fisher’s exact test

**Fig. 1:**
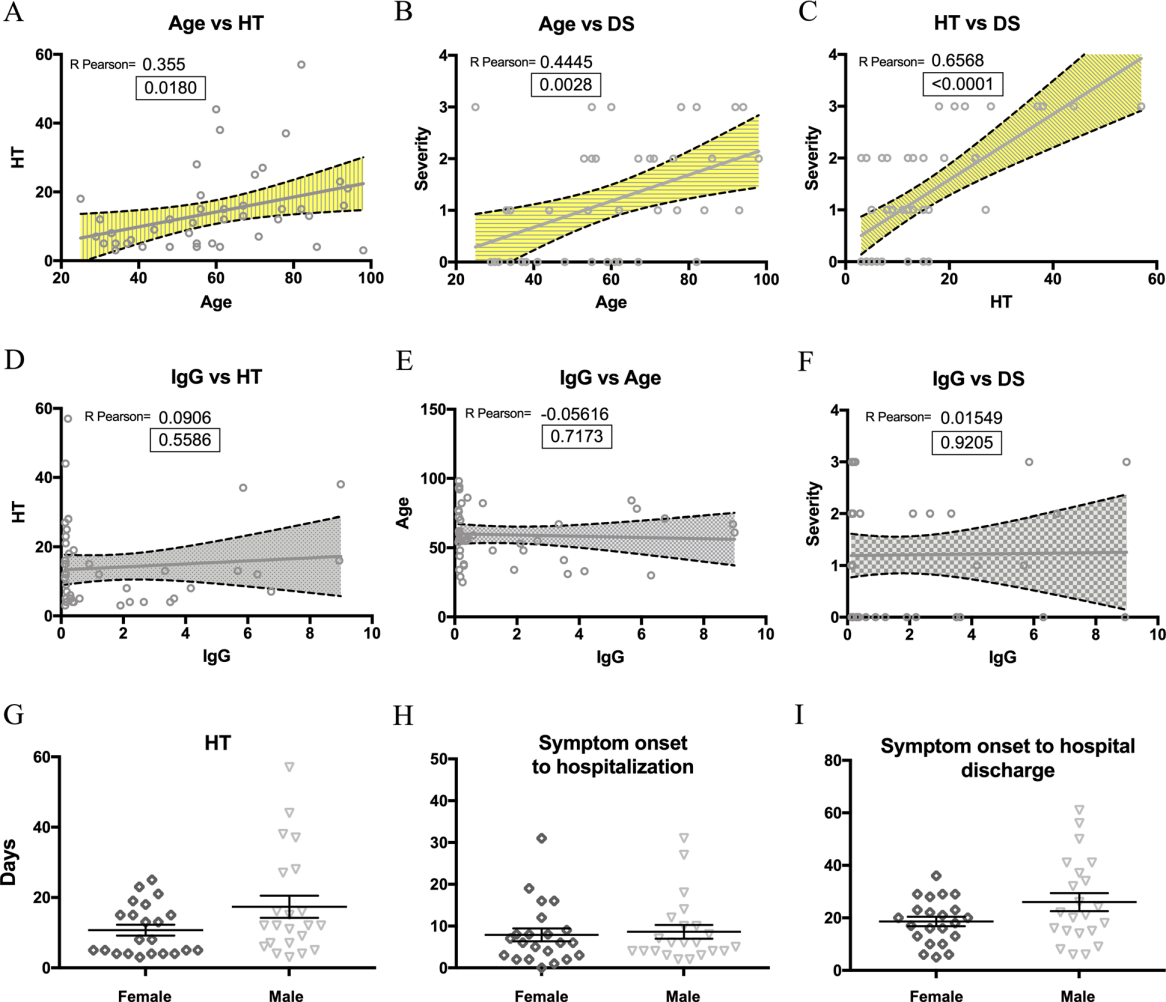
Correlative analysis between demographic and clinical parameters in the COVID-19 patient cohort. A positive correlation between age and HT (**A**), age and DS (**B**) or HT and DS (**C**) was measured by Person coefficient r (95% confidence interval) and two-tailed p-value analysis (indicated inside the square). Correlation analysis of SARS-CoV-2 -specific IgG with HT (**D**), age (**E**) or DS (**F**) measured by Person coefficient r (95% confidence interval) and two-tailed p-value analysis (indicated inside the square). Sex-matched analysis of HT (**G**), days from symptoms onset to HA (**H**), and days from symptom onset to hospital discharge (**I**); all data are expressed as mean of days ± S.E.M. In B, C and F, DS is indicated as following: 0 = mild, 1 = moderate, 2 = severe, 3 = critical.

By performing correlation analysis in our clinical dataset, we obtained a significant positive correlation between age and HT (R Pearson 0.35350 Fig. 1A) and, in agreement with the current literature^12,13^, we confirmed a positive association between age and DS (R Pearson 0.4445, Fig. 1B), and HT versus DS (R Pearson 0.6568, Fig. 1C) in our cohort.

The generation of IgG antibodies against SARS-CoV-2 proteins might represent an applicable parameter for COVID-19 patient stratification. Nevertheless, the parallel between SARS-CoV2 seropositivity and the clinical outcome is still a matter of investigation^14,15^. In our cohort, 34% of the patients (15/44 patients) showed positive IgG titer against the SARS-CoV-2 spike protein at the admission time (AT). However, no correlation between IgG positivity and HT (Fig. 1D), age (Fig. 1E), or DS (Fig. 1F) was evident.

Although it has been reported that COVID-19 mortality is higher in men than in women^16^, we did not observe major differences in the HT between females (50%) and males (50%) in our cohort study, with an HT mean of days 10.72 for female (1.52 SEM) and HT mean of days 17.36 for male (3.15 SEM) (Fig. 1G) and not even a significant variation in the timing from symptom onset to hospital admission (Fig. 1H) and hospital discharge (Fig. 1I).

**Fig. 2:**
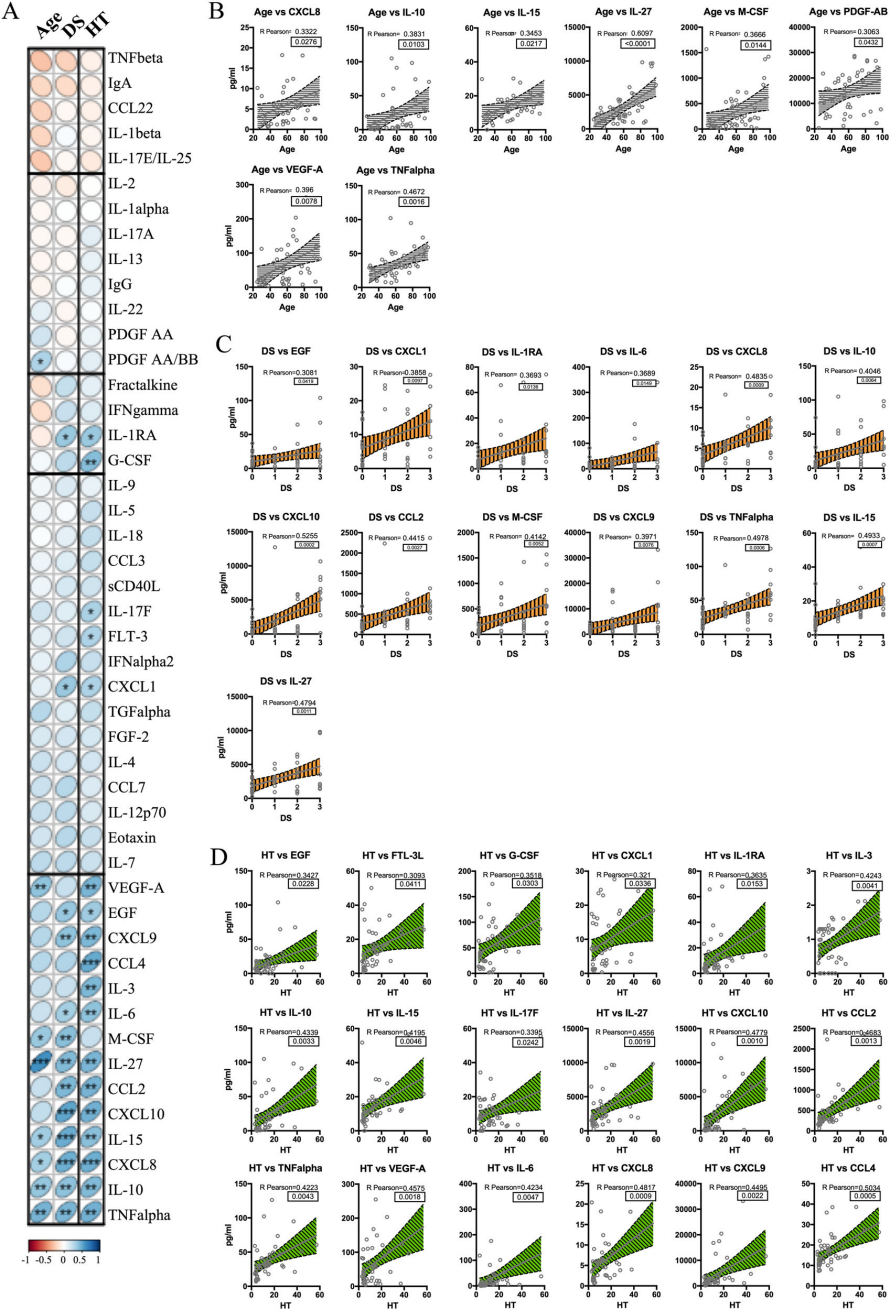
Cytokine correlation to age, HT, and DS in the COVID-19 patient cohort. Correlation matrix between cytokines and age, DS, and HT (**A**). The upward slope of the ellipses shows positive correlations in blue whereas downward ones show negative correlations in red. Color intensities and sizes of ellipses are proportional to the absolute value of the corresponding Pearson correlation coefficients (legend at the bottom side). The analytes are clustered according to the similarity of their correlation coefficients (horizontal black lines) using the “hclust” function of the R package “stats” according to the “ward.D2” method applied to ‘manhattan’ distances. Figure generated with the R package corrplot^32^. Individual cytokine details of Pearson’s correlation (95% confidence interval) of the indicated analytes with age (**B**, gray and horizontal lines), DS (**C**, orange and vertical lines 0 = mild, 1 = moderate, 2 = severe, 3 = critical) and HT (**D**, green and diagonal pattern) are indicated within each graph together with the two-tailed *p*-value analysis (boxed). *p < 0.05, **p < 0.01, ****p < 0.0001.

**Fig. 3:**
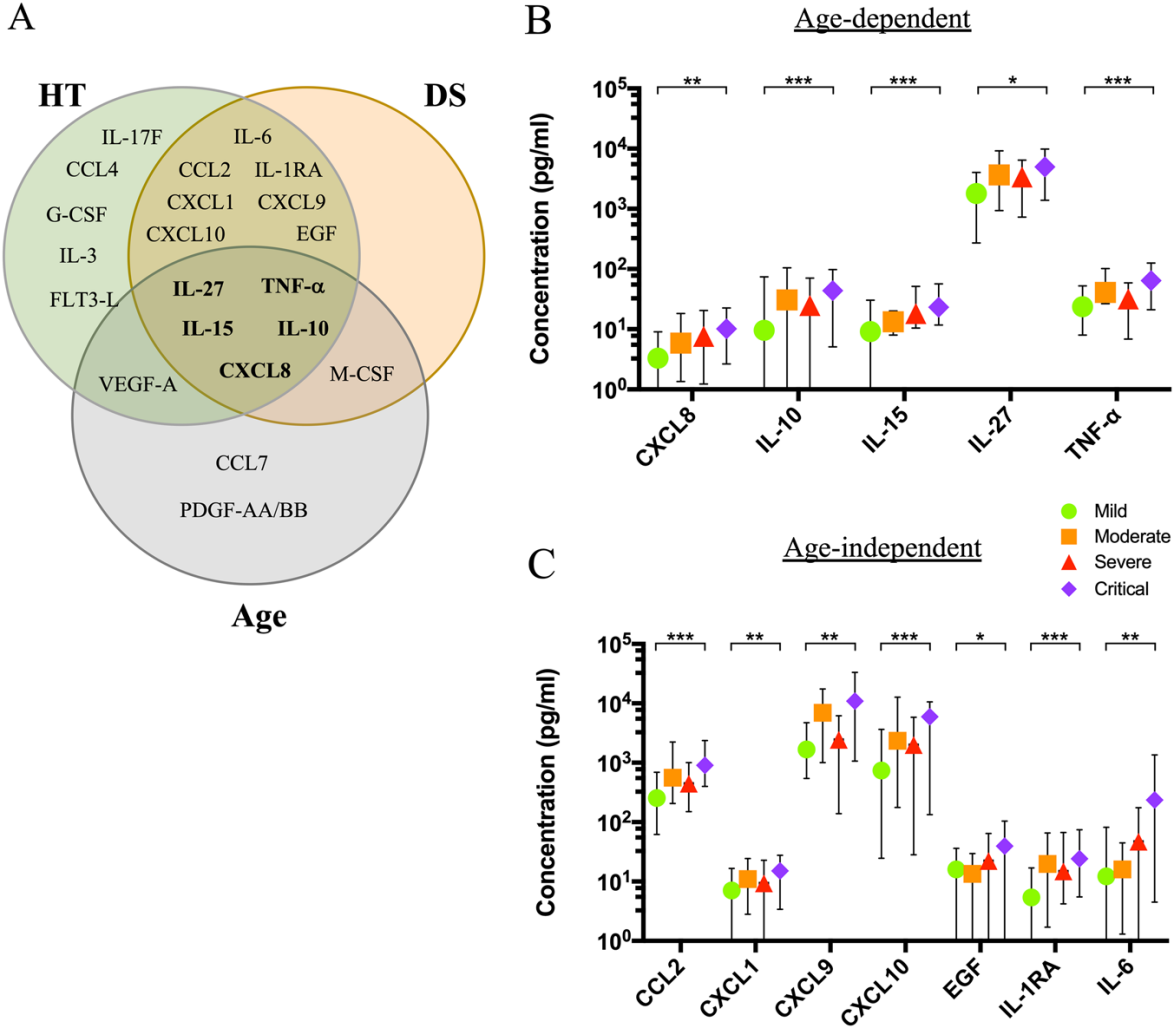
Cytokine profiles stratify COVID-19 patients. Venn diagram showing cytokines, chemokines, and growth factors as related to Age (*Gray*), HT (*Green*), and DS (*Orange*) (**A**). Graphs indicate the minimum (Min), maximal (Max), and mean plasma concentration values (pg/ml) of age-dependent (**B**) or age-independent (**C**) cytokines, chemokines, and growth factors of patients with the indicated DS score. The *P-value* (mild versus critical) has been calculated using a non-parametric Mann–Whitney test.

A properly-coordinated immune response represents a mandatory requirement for the clearance of SARS-CoV-2 infection^17^. Importantly, circulating factors play a crucial role in the immunopathology of SARS-CoV-2 infection and, in some cases, they might also tailor patient clinical path^18^. To outline the prevailing immune milieu in our cohort, we quantified cytokines and growth factors in patients’ plasma at admission time. To this aim, by multiplexed analysis, we concomitantly measured 48 circulating analytes and we performed correlation analysis between the plasma concentration of each analyte and HT, age, or DS, as defined by the correlation matrix (Fig. 2A). Here, the upward slope of the ellipses showed positive correlations (blue ellipses) while downward ones indicated negative correlations (red ellipses). Color intensities and sizes of ellipses are proportional to the absolute value of the corresponding Pearson correlation coefficients. Among all analytes, the correlogram revealed a distinctive pattern of cytokines showing a positive correlation with age (Fig. 2B), DS (Fig. 2C), or HT (Fig. 2D). On the other side, an additional set of cytokines unveiled no association with HT (Fig. 1S), age (Fig. 2S), or DS (Fig. 3S). The Venn diagram represents a unique set of cytokines that were differentially expressed in the three groups (Fig. 3A). As expected, the cytokine signatures associated with HT and DS were partially overlapping (12 out of 18 for HT, 12 out of 13 for DS). These shared cytokines include molecules that have been implicated in COVID-19 pathogenesis such as IL-1RA^19^, IL-6, CXCL10, CXCL8, IL-10, CCL2, CXCL9, and TNF-α^4,20^, as well as molecules that have not been associated to severity yet, such as IL-15, IL-27, and EGF. Interestingly, a specific subset of nine factors was selectively increased in older patients. Among these, we singled out a unique set of five cytokines - CXCL8, IL-10, IL-15, IL-27, and TNF-α - shared among the three variables. We also pointed out a defined cytokine trait (IL-6, CXCL9, IL-1RA, CXCL1, CXCL10, CCL2, EGF) of more severe COVID-19 patients, which is independent of age. To further associate the identified cytokine profiles to the clinical evaluation, the absolute plasma concentrations of the aforementioned age-dependent and age-independent cytokines were compared in patients with different disease severity, confirming that the cytokines were significantly up-regulated in critical cases, as compared to patients with mild disease (Fig. 3B–C).

**Fig. 4:**
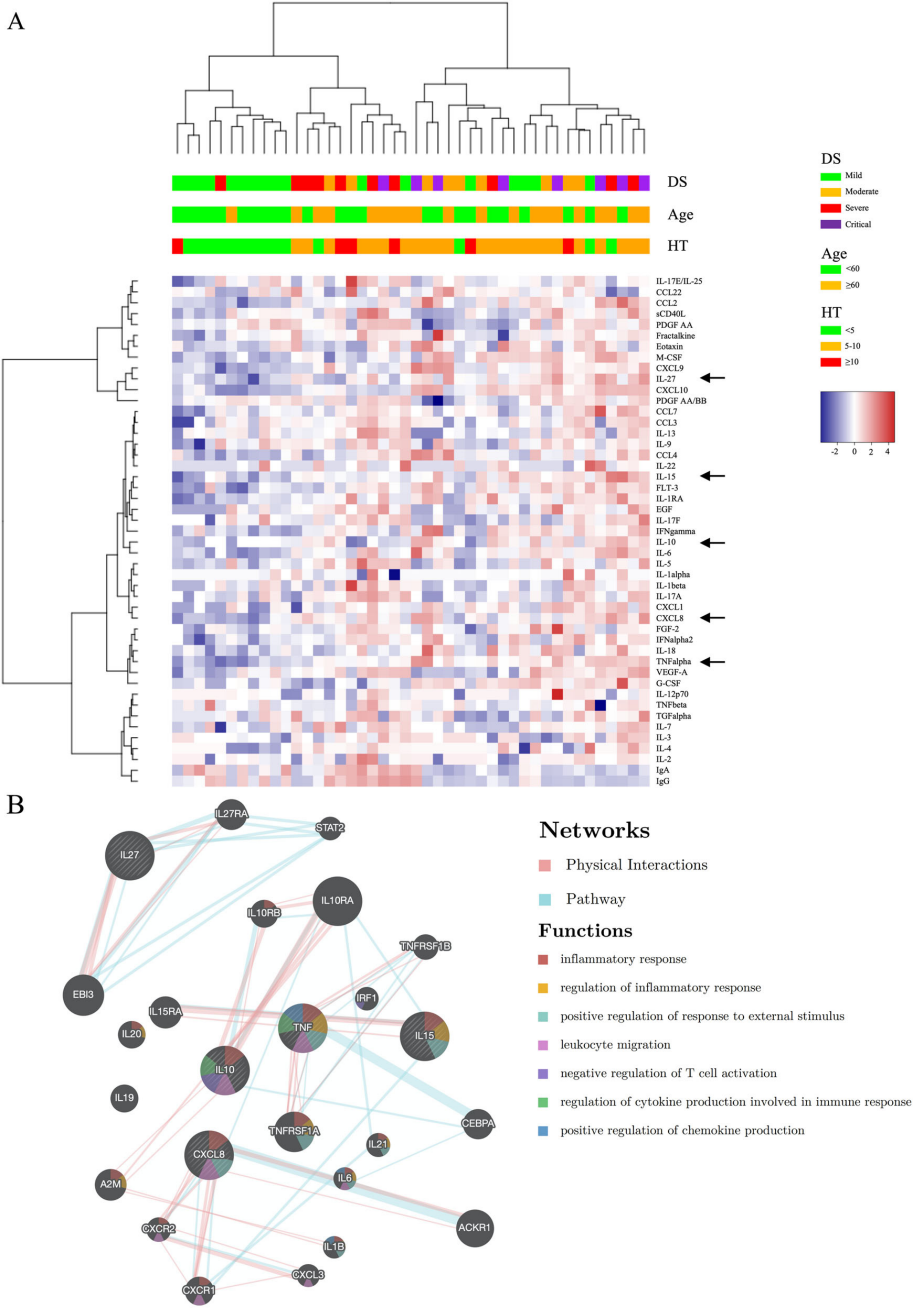
Cytokine clustering and functional analysis in COVID-19 patients. Heatmap represents an unsupervised clustering of the Luminex Assay analytes in 44 patients (every vertical line indicates one patient). On top of the severity, age, and HT clinical characteristics of each patient are reported as color codes according to the legend on the right. Clusterings were calculated using the “hclust” function of the R package “stats” according to the “ward.D2” method applied to “manhattan” distances and visualized through the ‘heatmap3’ package. Black arrows indicate the 5 cytokines correlating simultaneously with Age/HT/DS (**A**). Network integration for the 5 cytokines correlating simultaneously with Age/HT/DS, the connections were built by physical interactions (Red lines), and Pathway association (Green lines), main biological functions enrichment are listed (right) (**B**), analysis was performed using GeneMANIA algorithm.

We further assessed the correlation between all cytokines and the considered demographic/clinical parameters, by unsupervised clustering analysis (Fig. 4A). The heatmap and the clustering dendrogram confirmed the presence of specific cytokine profiles depending on DS, age, and HT. Moreover, the analysis demonstrated that analytes stratified in two main branches, grouped on the basis of concentration patterns. Intriguingly, 4 out of 5 of the cytokines (CXCL8, IL-10, IL-15, and TNF-α) shared among the three variables (DS, age, and HT) fell in a unique cluster, while IL-27 belongs to a different one, thus suggesting two independent activated networks in our patient cohort. The association of these proteins to different pathways has been validated through the biological network integration, considering physical interactions and pathway relations (Fig. 4B). We took advantage of the tool GeneMANIA^21^ that finds relationship among interested genes or proteins, but also with other genes that are related to input genes, using a very large set of functional association data. Association data include protein and genetic interactions, pathways, co-expression, co-localization, and protein domain similarity. Enrichment data revealed that CXCL8, IL-10, IL-15, and TNF-α interact with each other, but not with IL-27, thus reflecting different biological functions during the immune response.

**Fig. 5:**
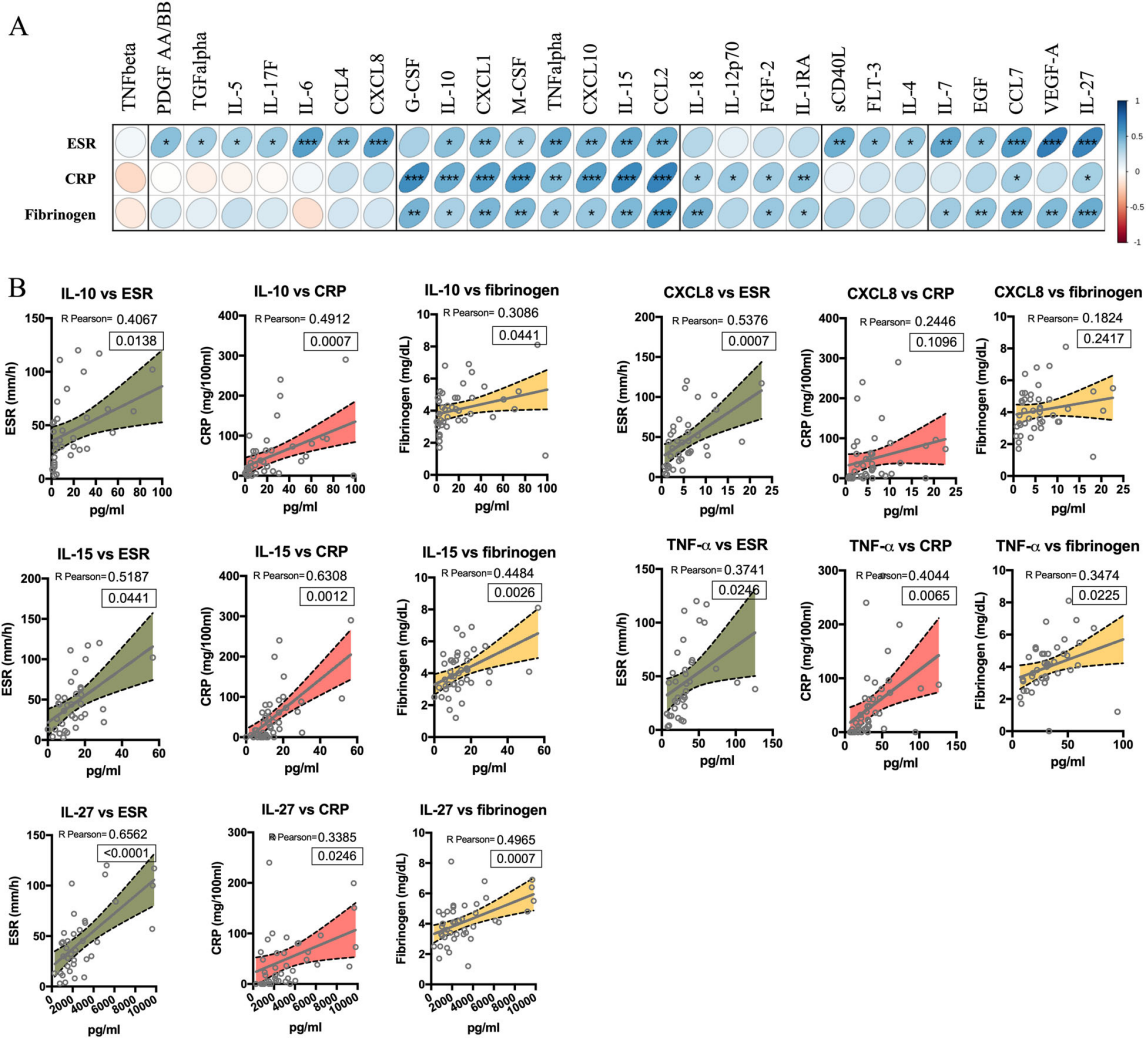
Clinical relevance of COVID-19-related cytokines. Correlogram showing a correlation between cytokines, chemokines, and growth factors - that have been shown to positively correlate with at least one of the three parameters (HT, age, and DS) - with the clinical features considered in our study (ESR, CRP, and fibrinogen) (**A**). Graphs show individual cytokines details of Pearson’s correlation (95% confidence interval) of all the analytes positively correlating with either ESR (green), CRP (red) amd fibrinogen (yellow) (**B**). Person coefficient r (95% confidence interval) and two-tailed p-value analysis (boxed) were calculated. *p < 0.05, **p < 0.01, ****p < 0.0001.

Next, we performed correlation analysis among cytokines that positively associated with DS, age, and HT with diagnostic parameters such as erythrocyte sedimentation rate (ESR), C-reactive protein (CRP), and fibrinogen concentration (Correlation Matrix Fig. 5A). Enlarged graphs (Fig. 5B) showed a positive correlation between ESR, CRP, and fibrinogen concentration and cytokines shared among DS, age, and HT (CXCL8, IL-10, IL-15, TNF-α, and IL-27).

**Fig. 6:**
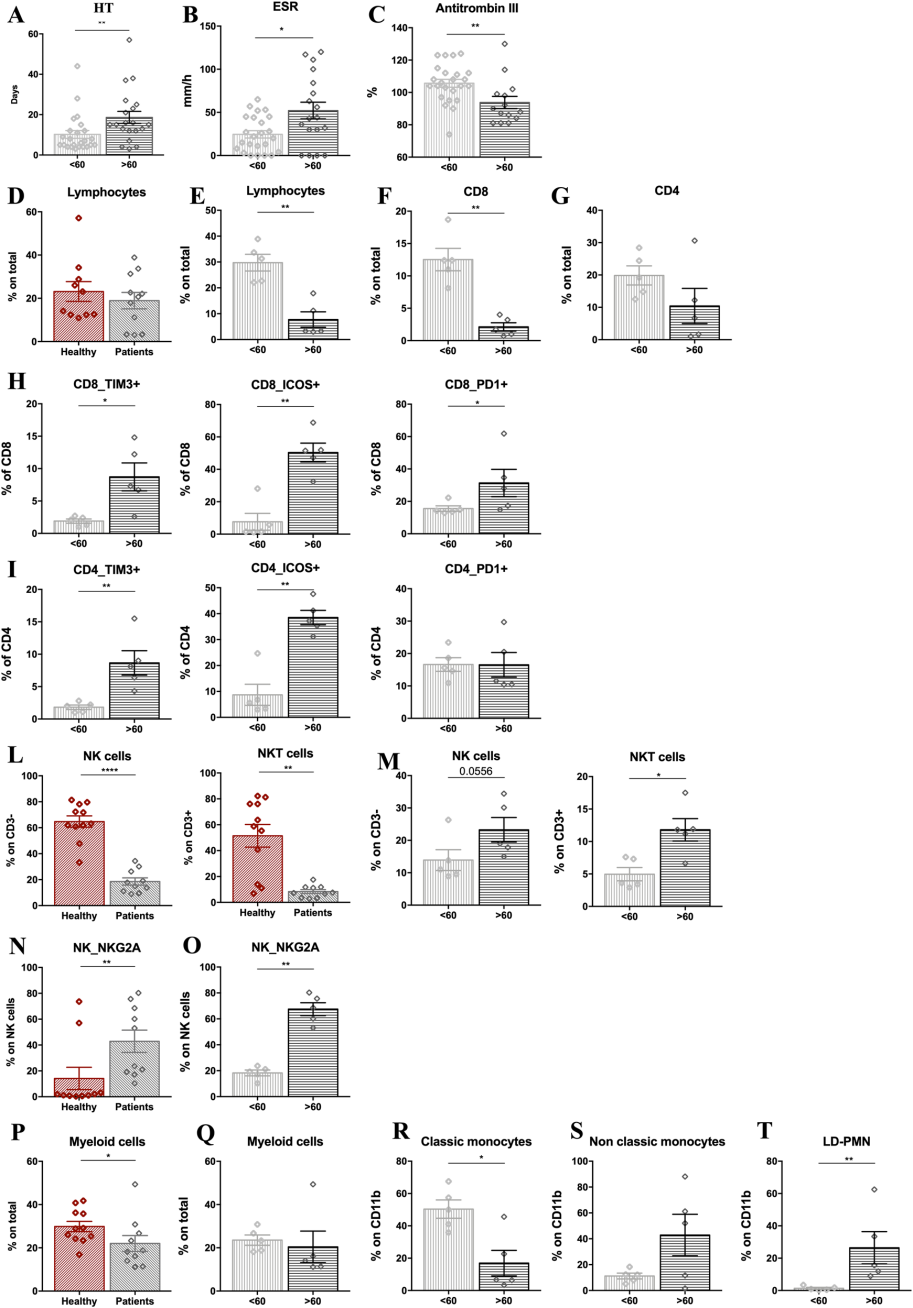
Age-dependent immune profiling of COVID-19 patients. Bar-charts showing HT, days **(A)**, ESR, mm/h (**B**) and antithrombin III, % (**C**) in younger (<60, light gray) or older (>60, dark gray) COVID-19 patients. Bar-charts showing T lymphocyte percentage (CD3^+^ T cells) in healthy (red), age-matched controls and COVID-19 patients (gray) (**D**), or in younger (<60, light gray) or older (>60, dark gray) COVID-19 patients (**E**). FACS analysis of immune cell subsets in younger (<60) and older (>60) COVID-19 patients. Bar-charts showing the Percentage of total CD8^+^ (**F**) or CD4^+^ **(G**) lymphocytes and the expression of TIM3, ICOS, and PD1 in younger (<60, light gray) or older (>60, dark gray) (**H–I**) COVID-19 patients; the percentage of NK and NKT cells in healthy age-matched controls and COVID-19 patients (**L**); the percentage of NKT and NK cells in younger (<60) and older (>60) COVID-19 patients (**M**); NKG2A expression in NK cells in healthy age-matched controls or COVID-19 patients (**N**) and in younger (<60) or older (>60) COVID-19 patients (**O**); the percentage of CD11b^+^ myeloid cells in healthy age-matched controls and COVID-19 patients (**P**) and in younger (<60) or older (>60) COVID19 patients (**Q**); the percentage of classical CD11b^+^CD14^high^CD16^−^ (**R**), and non-classical CD11b^+^CD14^l^^ow^CD16^+^ (**S**) monocytes, and LD-PMN CD11b^+^HLA-DR^lowneg^CD14^−^CD15^+^CD66b^+^ (**T**) in younger (<60) or older (>60) COVID-19 patients. Data are represented as mean of the percentage of parent population ± SEM. Gating strategy in Fig S6. Mann-Whitney test; *p < 0.05, **p < 0.01, ****p < 0.0001. Healthy *N* = 11, COVID-19 Patients = 10 (<60 *N* = 5, >60 *N* = 5).

Patients’ age has been appointed as a crucial determinant for the response to SARS-CoV-2, being older people generally at higher risk of severe illness^22,23^. In addition, it has been described that 80 and 90% of deaths have occurred in patients aged >70 years and >60 years in Korea and Italy, respectively^24^. In line with this evidence, our results identified a specific cytokine profile associated with COVID-19 in older patients. To further characterize patients’ immune responses in relation to age, we performed a detailed characterization of circulating immune cells in a subset of patients at the admission time (Fig. 6 and Fig. 4S), by performing multiparametric FACS analysis of peripheral blood cells. We stratified our cohort study by fixing the age of 60 as the cut-off for patient grouping. Of note, these 2 groups clearly differed in terms of clinical parameters, with age >60 patients having longer hospital stay HT (18.65 ± 2.96 S.E.M) as compared to the younger (10.21 ± 1.92 S.E.M) (Fig. 6A), higher ESR (Fig. 6B) and lower antithrombin III (Fig. 6C) at the admission time. Moreover, although we did not observe divergences in the total CD3^+^ lymphocyte counts in COVID-19 patients as compared to age-matched controls (Fig. 6D), a significant reduction in the total lymphocyte number appeared when our patients were stratified in >60> years old (Fig. 6E).

The flow cytometry analysis of peripheral blood cells showed that the reduction in total CD3^+^ T cells observed in older patients was retained in the CD8^+^ T cell subsets (Fig. 6F), with a similar trend in the CD4^+^ compartment (Fig. 6G). Additionally, in patients over 60 years, the level of expression of TIM3, ICOS, and PD1 molecules dramatically increased on both CD8^+^ (Fig. 6H) and CD4^+^ T lymphocytes (Fig. 6I), suggesting that T cells are characterized by an inactive-exhausted phenotype in this group. Consistently with previous reports^17^, we confirmed a general decrease in the count of NK T and also NK cells in patients, when compared to healthy subjects (Fig. 6L). However, but very interestingly, we detected an increased number of NK T cells in the >60 patients compared to the <60 ones, with a similar trend in the NK compartment (Fig. 6M). As previously reported^25^, NK cells in COVID-19 patients presented an exhausted phenotype, as defined by the expression of the higher expression of the inhibitory receptor NK group 2 member A (NKG2A), compared to healthy donors (Fig. 6N); but, remarkably, this higher NKG2A expression was specific of the over 60 groups (Fig. 6O). Finally, we detected a slight but significant reduction of the total CD11b^+^ events in patients compared to controls (Fig. 6P), but no age-related differences were observed (Fig. 6Q). Age-dependent differences were observed in terms of monocyte phenotype, being the classical monocytes (CD11b^+^/CD14^high^/CD16^−^) less represented in >60 patients (Fig. 6R) as compared to the non-classical monocyte (CD11b^+^/CD14^low^/CD16^+^ subset) (Fig. 6S). Moreover, we observed an expansion of a subset of granulocytic cells (CD11b^+^/HLA-DR^lowneg^/CD14^−^/CD15^+^/CD66b^+^) in this group, mostly resembling low-density polymorphonucleocytes (LD-PMN) (Fig. 6T) that have been described in sepsis and systemic inflammatory response syndrome, and that might play multiple immunomodulatory activities, including the suppression of T cell responses^26^.

Collectively, these sets of data suggest that in patients over 60 years old there is a specific immune signature characterized by the suppression of T cell responses and deregulated innate immunity. In this regard, the increased level of circulating IL-15 in severe patients, having a longer hospitalization, might nourish the expansion of NK cell subsets in aged people as compared to the younger; on the other side, the prolonged exposure of NK cells to the circulating IL-15 might be responsible for the reduction of their cytolytic activity, potentially triggering a deregulated exhausted phenotype of these cells in the >60 group^27^. We also confirmed an exhausted makeup of both CD4^+^ and CD8^+^ T lymphocytes in older patients; this could represent an additional suppressive mechanism, feed by the peculiar cytokine milieu that contributed to the inadequate immune response against the SARS-CoV-2 virus in aged patients. Among all cytokines, we identified IL-27 as the circulating factor that showed the best correlation coefficient with age (R Parson = 0.6097, Fig. 2B), is also associated with HT and DS (Fig. 2). In COVID-19 patients, IL-27 might be involved in the up-regulation of inhibitory receptors such as TIM3 and in skewing T cell responses. Moreover, IL-27 might modulate T cell activity by inducing IL-10 production^28^, which we identified to be also increased in patients in relation to age and DS. In turn, IL-10 might feed immunosuppressive circuits orchestrated by LD-PMN, that was expanded in older COVID-19 patients, suggesting the possible targeting of immunosuppressive checkpoints for novel therapeutic approaches. Two other cytokines of the shared signature, TNF-α, and CXCL8, were also suggestive of bolstered innate immune responses in older SARS-CoV-2 infected individuals. In line with this, anti-TNF trials have been recommended for COVID-19 patients who developed *acute respiratory distress syndrome* (ARDS) in the 2 days following hospital admission^29^. Consistently with available studies^30^, increased CXCL8 level in our cohort delineated a severe illness in patients and it might also represent a predictive biomarker of acute lung injury and ARDS in these patients^31^.

In conclusion, this study identified distinctive immunological features of COVID-19 patients that associate with age and are predictors of disease severity. Being our analysis performed at hospital admission, this study suggests novel markers that can be explored to identify novel criteria for COVID-19 patient stratification at hospital entry. In addition, the analyses revealed specific age-dependent immune signatures that may shed light on COVID-19 pathogenesis.

## Methods

### Participants, study design, and data collection

44 adult patients hospitalized at the COVID-19 center of the infectious diseases division (IDD) of the University Hospital of Padua, Italy, between 9.04.2020 and 5.05.2020 were enrolled in the study. All patients were diagnosed with COVID-19 with SARS-CoV-2 infection confirmed by real-time reverse transcription-polymerase chain reaction method (WHO guidelines). 12 SARS-CoV-2- negative age-matched participants were considered as a control group. All patients were classified into mild, moderate, severe, and critical cases based on results from chest imaging, clinical examination, and symptoms (WHO guidelines). In addition, patients were stratified into two groups by fixing the age of 60 as the cut-off for patient grouping.

Demographic, clinical, laboratory data were extracted from paper and electronic medical records using a standardized data collection form. Laboratory data included: complete blood count, ESR, CRP, coagulation profile, serum biochemical tests. Both Chest X rays and CT scans were performed for all COVID19 patients.

### Ethical commitment

The local ethics committee was notified about the study protocol. The study was performed according to the ethical guidelines of the Declaration of Helsinki (7th revision). All the patients gave their written informed consent and all analyses were carried out on anonymized data as required by the Italian Data Protection Code (Legislative Decree 196/2003) and the general authorization issued by the Data Protection Authority.

### PBMC isolation

Peripheral blood (PB) from enrolled controls and COVID-19 inpatients. PB was collected in EDTA tubes and stored at 4 °C prior to processing for PBMC isolation and plasma collection. Peripheral blood mononuclear cells (PBMC) were isolated by density-gradient sedimentation using Ficoll–Paque PLUS (GE Healthcare, Germany) according to the manufacturer’s protocol. Post-purification the isolated PBMC were cryopreserved in cell recovery media containing 10% DMSO (Gibco), supplemented with 90% heat-inactivated HyClone™ Fetal Bovine Serum (FBS; GE Healthcare, Germany) and stored in liquid nitrogen. Plasma was then carefully removed from the 2/3 of the top layer using a sterile serological pipette until the mononuclear cell interphase, portioned and aliquots were stored at −80 °C until the analysis.

### Flow cytometry

PBMC immune cell phenotyping was performed by custom set-up panels and BD Lyotubes by multiparametric FACS analysis. PBMC were thawed in RPMI 1640 with L-Glutamine (LONZA) medium with 2% FBS (GE Healthcare, Germany). 1 × 10^6^ PBMC were resuspended in 100 μl PBS with 2% FBS (FACS buffer) and stained with antibody cocktails at 4 °C in the dark. Following surface staining, cells were washed with FACS buffer and fixed in FACS buffer/1%PFA. After two final washes, cells were resuspended in 200 μl FACS buffer and acquired on a BD FACSCelesta™ Cell Analyzer (BD Biosciences, San Diego, CA). A list of antibodies used in the multicolor panels can be found in Figs. 5S–6S–7S–8S.

### Luminex assay

In total 48 analytes (sCD40L, EGF, Eotaxin, FGF-2, Flt-3 ligand, CX3CL1, G-CSF, GM-CSF, CXCL1, IFNα2, IFNγ, IL-1α, IL-1β, IL-1ra, IL-2, IL-3, IL-4, IL-5, IL-6, IL-7, CXCL8, IL-9, IL-10, IL-12 (p40), IL-12 (p70), IL-13, IL-15, IL-17A, IL-17E/IL-25, IL-17F, IL-18, IL-22, IL-27, CXCL10, CCL2, CCL7, M-CSF, CCL22, CXCL9, CCL3, CCL4, PDGF-AA, PDGF-AB/BB, CCL5, TGF-α, TNF-α, TNF-β, VEGF-A) were analyzed by Luminex assay (Millipore, Billerica, USA) in the plasma from controls and 44 patients. The diluted standard and quality control were used according to the manufacture’s instruction. The plate was read on Luminex 200™. Analysis was performed using xPONENT 3.1 software.

### Immunoglobulin testing

SARS-CoV-2 (Spike S1domain epitope) IgG and IgA detection were performed by ELISA Assay Kit from Euroimmun according to the manufacture’s instruction. (https://www.euroimmun.it/coronavirus-2019-ncov/).

### Data quantification and statistical analysis

Flow cytometry data were analyzed with BD FACSDiva™ (BD, Italy) and statistical analyses were done in Prism 8.4 (GraphPad, USA). Statistical analyses were carried out using packages of the R statistical software. Cluster analyses were performed using the “heatmap3” v1.1.7 package applying the ‘ward.D2” method on “manhattan” distances, which gives result values similar to the “hclust” function from the ‘stats’ package. The resulting clusterings were also used for grouping variables in the correlation plots. The Pearson correlation coefficients and the corresponding p-values were calculated using the “cor” and “cor.test” functions respectively from “stats” package. The correlation plots were generated with the “corrplot” v0.84 package^32^. Individual Pearson correlation test was graphed in Prism 8.4. Protein interaction and pathway interaction networks were performed using GeneMANIA software (https://genemania.org/). Data plotted were expressed as mean with standard error of mean (SEM). Data distribution was assessed by D’agostino-Pearson and Shapiro-Wilk normality test. Unpaired Nonparametric *t*-test or Krustal–Wallis test followed by *post hoc* Dunn’s multiple comparations was used to compare differences between groups. Differences were considered statistically significant at confidence levels *P < 0.05 or **P < 0.01.

## Supplementary information

Supplementary Figure legend

Fig Sup 1

Fig Sup 2

Fig Sup 3

Fig Sup 4

Fig Sup 5

Fig Sup 6

Fig Sup 7

Fig Sup 8

## Data Availability

The data supporting the findings of this study are available from the corresponding authors upon written request.
