# Supplementary material for: Age-severity matched cytokine profiling reveals specific signatures in Covid-19 patients
Source: Cell Death Dis. 2020 Nov 6;11(11):957. doi: 10.1038/s41419-020-03151-z (PMC7646225; doi:10.1038/s41419-020-03151-z)
Supplement: Supplementary file 1 — Supplementary Figure legend [file 41419_2020_3151_MOESM1_ESM.pdf]

<u>SUPPLEMENTARY MATERIALS</u>

Supplementary Materials Legends

Figure 1S
HT- cytokines
Correlation analysis of analytes and HT measured by Person coefficient r (95% confidence interval) and two-tailed p-value analysis (indicated inside the square).

Figure 2S
Age- cytokines
Correlation analysis of analytes and age measured by Person coefficient r (95% confidence interval) and two-tailed p-value analysis (indicated inside the square).

Figure 3S
DS- cytokines
Correlation analysis of analytes and DS measured by Person coefficient r (95% confidence interval) and two-tailed p-value analysis (indicated inside the square).

Figure 4S
Immune cell population in age-matched Covid-19 patients
Percentage of immune cell populations assessed by FACS analysis in healthy age-matched controls and COVID-19 patients (**A**), and in younger (<60) or older (>60) Covid-19 patients (**B**).

Figure 5S
Gating strategy for the identification of T cell subsets.

Figure 6S
Gating strategy for the identification of T cell exhaustion.

Figure 7S
Gating strategy for the identification of NK and B cells.

Figure 8S
Gating strategy for the identification of myeloid cell subsets.
